# Household Air Pollution and Blood Pressure, Vascular Damage, and Subclinical Indicators of Cardiovascular Disease in Older Chinese Adults

**DOI:** 10.1093/ajh/hpab141

**Published:** 2021-09-10

**Authors:** Thirumagal Kanagasabai, Wuxiang Xie, Li Yan, Liancheng Zhao, Ellison Carter, Dongshuang Guo, Stella S Daskalopoulou, Queenie Chan, Paul Elliott, Majid Ezzati, Xudong Yang, Gaoqiang Xie, Frank Kelly, Yangfeng Wu, Jill Baumgartner

**Affiliations:** 1 Institute for Health and Social Policy, and Department of Epidemiology, Biostatistics and Occupational Health, McGill University, Montreal, Quebec, Canada; 2 Peking University Clinical Research Institute, Peking University Health Science Center, Beijing, China; 3 Department of Epidemiology and Biostatistics, and MRC Centre for Environment and Health, School of Public Health, Imperial College London, London, UK; 4 Fu Wai Hospital and Cardiovascular Institute, Chinese Academy of Medical Sciences, Beijing, China; 5 Department of Civil and Environmental Engineering, Colorado State University, Fort Collins, Colorado, USA; 6 Department of Cardiology,Yuxian Hospital, Yuxian, Shanxi, China; 7 Department of Medicine, Division of Internal Medicine and Division of Experimental Medicine, McGill University, Montreal, Quebec, Canada; 8 Department of Building Science, Tsinghua University, Beijing, China; 9 Environmental Research Group, MRC Centre for Environment and Health, School of Public Health, Imperial College London, London, UK

**Keywords:** arterial stiffness, atherosclerosis, blood pressure, fine particulate matter, hypertension, plaques, solid fuel

## Abstract

**Background:**

Limited data suggest that household air pollution from cooking and heating with solid fuel (i.e., coal and biomass) stoves may contribute to the development of hypertension and vascular damage.

**Methods:**

Using mixed-effects regression models, we investigated the associations of household air pollution with blood pressure (BP) and vascular function in 753 adults (ages 40–79 years) from 3 diverse provinces in China. We conducted repeated measures of participants’ household fuel use, personal exposure to fine particulate air pollution (PM_2.5_), BP, brachial–femoral pulse wave velocity (bfPWV), and augmentation index. Ultrasound images of the carotid arteries were obtained to assess intima–media thickness (CIMT) and plaques. Covariate information on sociodemographics, health behaviors, 24-h urinary sodium, and blood lipids was also obtained.

**Results:**

Average estimated yearly personal exposure to PM_2.5_ was 97.5 µg/m^3^ (SD: 79.2; range: 3.5–1241), and 65% of participants cooked with solid fuel. In multivariable models, current solid fuel use was associated with higher systolic (2.4 mm Hg, 95% CI: −0.4, 4.9) and diastolic BP (1.4 mm Hg, 95% CI: −0.1, 3.0) and greater total area of plaques (1.7 mm^2^, 95% CI: −6.5, 9.8) compared with exclusive use of electricity or gas stoves. A 1 − ln(µg/m^3^) increase in PM_2.5_ exposure was associated with higher systolic (1.5 mm Hg, 95% CI: 0.2, 2.7) and diastolic BP (1.0 mm Hg, 95% CI: 0.4, 1.7) and with greater CIMT (0.02 mm, 95% CI: 0.00, 0.04) and total area of plaques (4.7 mm^2^, 95% CI: −2.0, 11.5). We did not find associations with arterial stiffness, except for a lower bfPWV (−1.5 m/s, 95% CI: −3.0, −0.0) among users of solid fuel heaters.

**Conclusions:**

These findings add to limited evidence that household air pollution is associated with higher BP and with greater CIMT and total plaque area.

## Graphical Abstract

**Figure:**
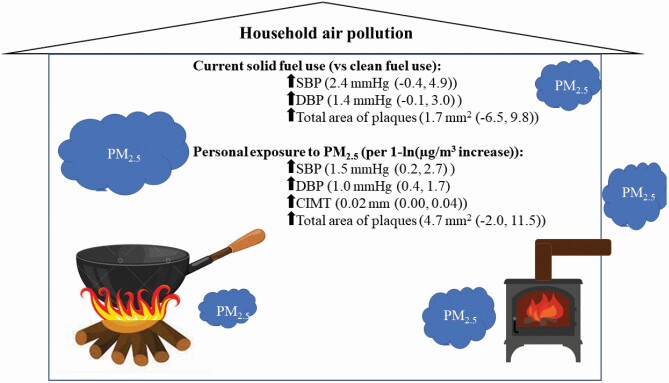

High blood pressure (BP) is the leading cause of death, responsible for 10.8 million premature deaths in 2019 worldwide.^1^ In China, 250 million people live with hypertension, which is responsible for 24% of China’s deaths and 14% of disability-adjusted life years.^2^ Urban air pollution shows strong and consistent associations with high BP and the development of cardiovascular disease in studies mostly conducted in high-income cities.^3,4^ Less understood is whether household air pollution from biomass and coal (i.e., solid fuel) stoves also contributes to hypertension and vascular damage, their underlying preclinical conditions, and the exposure–response relationships between them.^5^

Over 2.5 billion people globally and 600 million Chinese^3^ use solid fuel cookstoves and heaters that emit air pollution into homes and communities. Several cohort studies indicate that the primary use of a solid fuel stove increases the risk of mortality and nonfatal cardio-respiratory events by 7%–19%.^5^ These studies are supported by analyses showing higher BP and greater carotid intima–media thickness (CIMT) in women using wood-burning cookstoves,^6–8^ and that switching to less-polluting stoves lowers BP.^9^ However, despite widespread exposure to household air pollution, there remain very few exposure–response studies^10^ and even fewer that included men,^5^ which form a significant knowledge gap for policy.

Leveraging repeated measurements from the INTERMAP China Prospective (ICP) study,^11^ a geographically diverse cohort in rural and peri-urban villages, we investigated associations of estimated yearly exposure to fine particulate matter <2.5 microns (PM_2.5_) with the severity of arteriosclerosis and vascular disease, as measured by BP, arterial stiffness, CIMT, and carotid plaque features. We also assessed whether adults using solid fuel stoves at present and over the previous 2 decades had greater vascular damage than adults using only gas or electric (i.e., clean fuel) stoves.

## METHODS

### Study population

The ICP Study is an ongoing longitudinal study to investigate the nutritional and environmental risk factors for cardiovascular disease, including BP and vascular function.^11^ It is an extension of the INTERMAP study, a 1996 investigation of nutrient intake and BP in 839 adults aged 40–59 years that lived in 17 villages in northern (Beijing and Shanxi) and southern (Guangxi) China.^12^ In 2015, we recontacted 680 of the surviving INTERMAP study participants and enrolled 574 into the ICP Study (participation rate: 84%).^11^ An additional 208 adults ages 40–59 years (i.e., same ages as INTERMAP participants in 1996) were randomly selected from village lists and enrolled into the study (participation rate: 86%) to ensure sufficient sample size as some INTERMAP participants had died or moved away. Participants lived in rural and peri-urban villages that were within 20–30 km of urban areas, and provided written informed consent. Study protocols received ethics committee approvals from all investigator institutions.

### Data collection

Participants attended village clinics on up to 7 occasions during a total of 6 data collection campaigns across our study sites that took place between August 2015 and November 2016. We conducted at least 1 winter and summer campaign in the northern sites to capture seasonal differences, which resulted 1 full campaign in Guangxi and 2 full campaigns in Beijing and Shanxi. A full campaign consisted of 2 days of data collection, including repeated measurements of BP. During the first clinic visit in each campaign, staff verbally administered individual and household questionnaires to participants. During the second and third clinic visits in each campaign, we repeatedly measured participants’ anthropometrics, exposure to air pollution, BP, and arterial stiffness, and collected 24 hours urine for biochemistry. Ultrasound images of the carotid arteries and whole blood samples were obtained once in June (Shanxi), September (Beijing), and November (Guangxi) of 2016 (Supplementary Figure S1 online). The images were collected at the same time as other study measurements in Beijing and Guangxi, and in a separate third reduced data collection campaign in Shanxi where BP and surveys were also conducted. Details about measurements are provided below and published in a study protocol.^11^

### Current and historical household fuel use and intensity of use

We developed visual libraries of stoves and fuels for each site that were used to collect information on both current and historical fuel use during the first clinic visit. For each stove pictured, participants indicated whether they had used it during the past 20 years and, if so, answered questions about the years (in 5-year intervals) and frequency of use, stove location in the home, and fuel. Stove-fuel categories were combined with reported use data to construct fuel-based exposure metrics (Table 1). Cookstoves refer to devices used for household cooking or water boiling activities, whereas heating stoves are those primarily used for space heating during winter.^13^

**Table 1.** Household air pollution exposure variables and their descriptions^a^

Variable	Description
Household fuel use and intensity of use	
1.Current fuel for cooking	Any use of solid fuel cookstoves Exclusive use of clean fuel cookstoves (reference)
2.Current fuel for heating (northern China)	Any use of solid fuel heating stoves Exclusive clean fuel heating stoves (reference) No heating stoves^b^
3.Current intensity of indoor solid fuel stove use	Number of indoor solid fuel stove-use days in the past year, continuous
4.Long-term intensity of indoor solid fuel stove use	Number of indoor solid fuel stove-use years in the past 20 years, continuous
Exposure to air pollution	
5.Seasonal personal exposure to PM_2.5_ (µg/m^3^)	Gravimetric analysis of 2 consecutive 24-hour personal PM_2.5_ measurements collected in the heating or nonheating season, continuous. Repeated measures were used in the analysis.
6.Yearly personal exposure to PM_2.5_ (µg/m^3^)	Time-weighted average of season-specific measurements of PM_2.5_ exposure based on the assumption of 7 nonheating months and 5 heating months, continuous. Time weighted measures were used in the analysis.

^a^Clean fuel stoves included those powered by gas or electricity and solid fuel stoves included those powered by coal, wood, crop residues, or other forms of biomass.

^b^This group was excluded from the analysis given the small sample size.

### Personal exposure to air pollution

We measured personal exposure to PM_2.5_ for up to 96 hours in 2 seasons to estimate seasonal and yearly exposures (Table 1). Participants wore waist packs with air samplers that collected PM_2.5_ on PTFE filters. In a random 80% subsample, we monitored compliance in wearing the air samplers by placing pedometers (HJ-321 Tri-Axis, Omron, Japan) inside the waist packs. Filters were analyzed for mass at the Wisconsin State Laboratory of Hygiene. Details on PM_2.5_ measurement are published elsewhere.^11,14^

### Cardiovascular outcomes

We assessed 9 outcomes that reflect different domains of vascular disease: (i) brachial systolic and (ii) diastolic BP, representative of systemic vascular resistance; (iii) brachial–femoral pulse wave velocity (bfPWV), a direct measure of central and peripheral arterial stiffness, (iv) augmentation index (AIx), which measures wave reflection and indirectly measures arterial stiffness; (v) CIMT, a marker associated with systemic atherosclerosis; (vi) total area of plaques in the carotid artery, a measure of the presence and severity of carotid atherosclerosis; and (vii) plaque grayscale median (GSM), where lower GSM indicates greater instability.^15–18^

Following at least 5-min of quiet rest, BP was measured on the participant’s right arm at least 3 times using an automated oscillometric device (Omron HEM-907). Up to 5 measurements were taken if the difference between the last 2 was >5 mm Hg. BP was taken on 2 consecutive days during each measurement campaign, except for Shanxi’s third campaign which had only 1 day of BP measurement due to study logistics. The average of the last 2 measurements from consecutive days of each campaign was used for statistical analysis, preserving season-specific BP and arterial stiffness variations. Hypertension was defined as use of antihypertension medication, or systolic (≥140 mm Hg) and/or diastolic (≥90 mm Hg) BP. Up to 6 measurements, 3 per campaign, of bfPWV and AIx (Vicorder, Smart Medical, UK) were taken in the supine position with cuffs around the participant’s right upper arm and right upper thigh.

Measurements of CIMT and carotid artery plaques were conducted by a single technician in B-mode ultrasonography using a portable Mindray Z6 Ultrasound and 7–10 MHz linear array transducer. At least 3 images of the far wall of 3 10-mm segments of the bilateral carotid artery were obtained for both arteries, i.e., 18 images total.^18^ A clinician analyzed each image offline using an automated software (Carotid Analyzer Vascular Research Tools 6, Medical Imaging Applications, Coralville) to assess CIMT, total area of plaques, and GSM, and the average of all measurements was used for statistical analysis. A plaque was defined as a localized thickness of CIMT ≥1.5 mm or a focal raised lesion of ≥0.5 mm, and at least 3 images were obtained. Plaques were measured by manually tracing the perimeter and computing their cross-sectional area. Blood pressure, ultrasound measurements, and image-based analyses were conducted by staff who were blinded to the participants’ exposure status.

### Covariates

Staff administered individual and household questionnaires in each campaign to obtain information on household income and sociodemographics and assessed individual cardiovascular risk factors. Serum specimens were collected once and processed in a centralized testing facility for lipids, and 24-hour urine samples were collected twice in each campaign and analyzed for sodium. Anthropometrics were measured in each campaign using a height ruler attached to a mechanical scale and SECA measuring tapes.

### Statistical analysis

Mixed-effects regression models with participant- and village-specific random intercepts were used to estimate associations between household air pollution, measured by fuel use and exposure to PM_2.5_, and outcomes with repeated measures (BP, bfPWV, AIx). These models account for the lack of independence between repeated measurements in the same participant and between participants in the same village. Cross-sectional models with village-specific random intercepts were conducted for outcomes with single measurements (CIMT, total plaque area, GSM). Known and suspected confounders included in the final models were age, age, sex, household income, waist circumference, height (AIx, bfPWV), smoking status, secondhand smoke exposure, alcohol consumption, physical activity, 24-h urinary sodium (BP, bfPWV, AIx), time of BP measurement (PM_2.5_ models), and total/HDL cholesterol ratio.^19^ Waist circumference and body mass index were highly correlated (*r* = 0.8); thus, we adjusted only for waist circumference which was a stronger confounder. We did not adjust for outdoor temperature because it was strongly correlated with PM_2.5_. We also did not adjust for previous diagnosis of diabetes or hypertension in the main analysis given that they could be confounders or along the causal pathway.

Seasonal measurements of PM_2.5_ were time-weighted (7 nonheating and 5 heating months) to estimate average yearly exposure for models predicting CIMT and plaque features. Homogeneity of variance and normality assumptions were evaluated by residual vs. fitted plots and QQ plots. Data were inspected to determine if participants with missing variables were systematically different from rest of the study population based on other information collected, and found that the missing variables appeared to be missing at random. We handled missing variables with multiple imputation for income (*n* = 87), lipids (*n* = 107), urinary sodium (*n* = 26), waist circumference (*n* = 16), and body mass index (*n* = 17) (Supplementary Table S1 online). Median village- and campaign-specific values were used for participants missing the time of BP measurement (*n* = 29).

We assessed effect modification for variables previously shown to modify air pollution-vascular associations including by sex, age, antihypertensive medication use, and geographic region for all outcomes and by season for BP and arterial stiffness. Sensitivity analyses included (i) limiting fuel use analyses to indoor stoves; (ii) excluding participants with self-reported diabetes, which could be a confounder or along the causal pathway; (iii) adjusting for heating fuel in models with cooking fuel as the exposure and vice versa; and, (iv) adjusting for hypertension status in models of arterial stiffness, CIMT, and plaque features since high BP could be a confounder or along the causal pathway. Statistical analyses were conducted in R (www.r-project.org, v. 1.2.1335).

## RESULTS

### Characteristics of the study population

At the time of the first survey, participants were 40–79 years old (median = 63 years), 55% female, and 23% were current tobacco smokers (Table 2). Exclusive users of clean fuel, on average, had higher education and income, were more likely to smoke, less likely to regularly engage in physical activity, and more likely to report taking antihypertensive medication and have diabetes compared with solid fuel users. Current exclusive users of clean fuel had lower personal exposures to PM_2.5_ than current users of solid fuel cookstoves [mean (SD): 91.7 (63.0) vs. 100.6 µg/m^3^ (86.4)].

**Table 2.** Characteristics of study participants by current cooking fuel use (*n* (%) or mean (SD))

	Exclusive use of clean fuel	Any use of solid fuel	All	
Characteristic	(*n* = 266)	(*n* = 487)	(*n* = 753)	*P* value
Age (years)	61.7 (9.3)	63.4 (8.2)	62.8 (8.6)	<0.01
Sex (% female)	142 (53.4%)	274 (56.3%)	416 (55.2%)	0.22
Province				
Beijing	73 (27.4%)	173 (35.5%)	246 (32.7%)	<0.01
Guangxi	68 (25.6%)	155 (31.8%)	223 (29.6%)	
Shanxi	125 (47.0%)	159 (32.6%)	284 (37.7%)	
Highest educational attainment				
No formal education	37 (13.9%)	77 (15.8%)	114 (15.1%)	<0.01
Primary school	104 (39.1%)	208 (42.7%)	312 (41.4%)	
Early high school, college, or above	125 (47.0%)	202 (41.5%)	327 (43.4%)	
Yearly household income (CYN)				
<20,000	118 (44.4%)	232 (47.6%)	350 (46.5%)	0.17
≥20,000	148 (55.6%)	255 (52.4%)	403 (53.5%)	
Occupation				
Agricultural	152 (57.1%)	286 (58.7%)	438 (58.2%)	0.10
Retired or not currently employed	87 (32.7%)	166 (34.1%)	253 (33.6%)	
Nonagricultural	27 (10.2%)	35 (7.2%)	62 (8.2%)	
Tobacco smoking				
Never	150 (56.4%)	302 (62.0%)	452 (60.0%)	0.12
Past	48 (18.0%)	78 (16.0%)	126 (16.7%)	
Current	68 (25.6%)	107 (22.0%)	175 (23.2%)	
Secondhand smoke exposure				
Never	159 (59.8%)	270 (55.4%)	429 (57.0%)	0.35
Past	43 (16.2%)	80 (16.4%)	123 (16.3%)	
Current	64 (24.1%)	137 (28.1%)	201 (26.7%)	
Alcohol consumption (past year)				
Never	167 (62.8%)	293 (60.2%)	460 (61.1%)	0.19
Occasional (<1 drink per week)	47 (17.7%)	107 (22.0%)	154 (20.5%)	
Regular (≥1 drink per week)	52 (19.5%)	87 (17.9%)	139 (18.5%)	
Physical activity (frequency in past 3 months)				
None	66 (24.8%)	82 (16.8%)	148 (19.7%)	<0.01
≤2 times per week	62 (23.3%)	107 (22.0%)	169 (22.4%)	
≥3 times per week	138 (51.9%)	298 (61.2%)	436 (57.9%)	
Hypertension (% yes)	141 (53.0%)	252 (51.7%)	393 (52.2%)	0.96
Current use of antihypertensive medication (% yes)	107 (40.2%)	174 (35.7%)	281 (37.3%)	0.33
Clinician-diagnosed diabetes (% yes)	40 (15.0%)	51 (10.5%)	91 (12.1%)	<0.01
Waist circumference (cm)	88.2 (9.7)	86.7 (10.0)	87.2 (9.9)	<0.01
Body mass index (kg/m^2^)	25.6 (3.5)	25.1 (3.9)	25.3 (3.8)	<0.01
Height (cm)	158.9 (9.5)	158.4 (8.5)	158.6 (8.9)	0.48
Mean 24-hour urinary sodium excretion (mmol/day)	168 (80)	174 (82)	171 (81)	0.02
Total cholesterol (mmol/l)	5.0 (1.2)	5.0 (1.1)	5.0 (1.1)	0.22
LDL (mmol/l)	3.0 (1.0)	3.0 (0.9)	3.0 (0.9)	0.08
HDL (mmol/l)	1.3 (0. 4)	1.3 (0.3)	1.3 (0.3)	0.68
Total/HDL cholesterol ratio	4.1 (3.6)	3. 9 (1.0)	4.0 (2.3)	0.74

Abbreviations: CYN, Chinese Yuan; ; HDL, high-density lipoproteins; , Hypertension = current use of antihypertensive medication, systolic (≥140 mm Hg) or diastolic (≥90 mm Hg) blood pressure; Physical activity = exercise and farm-based physical activity domains. Characteristics of our study population are provided by province and sex in Supplementary Table S2 online, and variables with missing data are provided in Supplementary Table S1 online.

Over half (52%) of the participants had hypertension, 72% of whom reported taking antihypertensive medication. Mean BP and brachial–femoral PWV were higher in winter (Table 3). Blood pressure (*P* = <0.01) and AIx (*P* = <0.01), but not bfPWV (*P* = 0.16), differed across provinces. CIMT was greater in northern participants compared with those in Guangxi (*P* = 0.02). Nearly 75% of participants with CIMT measurement had at least 1 carotid plaque (*P* < 0.01). Sex and province-specific demographics are provided in Supplementary Table S2 online. During the study period, 7 participants reported diagnoses of incident coronary heart disease and 5 reported having a stroke.

**Table 3.** Descriptive statistics for blood pressure and vascular outcomes and exposure to household air pollution, by study site and campaign (mean (SD), median [min, max], or *n* (%))

	Shanxi			Beijing		Guangxi
	Campaign 1	Campaign 2	Campaign 3	Campaign 1	Campaign 2	Campaign 1
	Aug-15	Nov-15	Jun-16	Dec-15	Sep-16	Nov-16
	(*n* = 277)	(*n* = 218)	(*n* = 245)	(*n =* 237)	(*n* = 217)	(*n* = 223)
Blood pressure and vascular outcomes						
Systolic blood pressure (mmHg)	125.9 (16.2)	136.9 (18.0)	130.2 (17.8)	138.9 (16.5)	131.0 (16.7)	133.6 (18.4)
Diastolic blood pressure (mmHg)	70.7 (9.7)	75.5 (10.4)	73.7 (10.9)	79.1 (10.7)	76.1 (10.5)	72.5 (11.0)
Brachial–femoral pulse wave velocity (m/s)	17.0 (6.9)	17.1 (5.7)		17.9 (7.0)	16.9 (5.1)	18.0 (6.7)
Augmentation index (%)	25.7 (6.9)	24.4 (5.7)		23.9 (6.1)	25.5 (5.7)	23.4 (6.4)
Carotid intima–media thickness (CIMT, mm)			0.7 (0.1)		0.8 (0.2)	0.7 (0.1)
Total area of plaques (mm^2^)			56.1 (59.6)		26.2 (37.2)	37.1 (48.8)
Grayscale median (mean of all plaques)			74.0 (41.4)		53.5 (37.4)	62.9 (44.2)
Exposure to household air pollution						
Current cookstove use						
Any use of solid fuel	159 (56.0%)			173 (70.3%)		155 (69.5%)
Exclusive use of clean fuel	125 (44.0%)			73 (29.7%)		68 (30.5%)
Current heating stove use						
Any use of solid fuel	213 (75.0%)			222 (90.2%)		
Exclusive use of clean fuel	55 (19.4%)			21 (8.5%)		
No heating stoves	16 (5.6%)			3 (1.2%)		
Current intensity of indoor solid fuel stove use (stove-use days per year)	268.1 (228.3)			204.4 (186.4)		275.1 (217.4)
Long-term (20 y) intensity of indoor solid fuel use (stove-use years)	20.0 [0.6, 33.3]			14.8 [0.8, 52.5]		15.0 [0.0, 35.0]
Personal exposure to PM_2.5_ (µg/m^3^)	103.3 (90.5)	147.0 (110.4)		127.6 (82.0)	74.8 (63.1)	59.3 (33.1)
Time-weighted personal exposure to PM_2.5_ (µg/m^3^)			101.5 (73.9)		89.9 (47.7)	59.3 (33.1)

Abbreviations: CCA, common carotid artery; ECA, external carotid artery; ICA, internal carotid artery; PM_2.5_, fine particulate matter <2.5 µm. Survey data indicated no major change in fuel or stove use between campaigns, such that the same fuel use exposure variables were used for each season in the repeated measures analysis.

### Exposure to solid fuel stove use and PM_2.5_

Most participants cooked with solid fuel (65%), and 82% of northern participants heated with solid fuel (Table 3). On average, participants reported 249 (SD: 214) indoor solid fuel “stove-use days” in the previous year, with greater intensity of use in Shanxi and Guangxi. Long-term (previous 20 years) intensity of indoor solid fuel stove use was greatest in Shanxi [mean (SD): 18.9 stove-use years (6.5) vs. 15.5 (8.5) in Beijing and 14.4 (8.5) in Guangxi]. Average estimated yearly exposures to PM_2.5_ were 97.5 µg/m^3^, which is ~10 times the WHO’s PM_2.5_ guideline (10 µg/m^3^). We observed a moderately high correlation between seasonal and estimated yearly average exposure to PM_2.5_ among participants with air pollution exposure assessment in more than 1 campaign (*r* = 0.78).

### Associations of solid fuel use with blood pressure and vascular function

**Figure 1.**
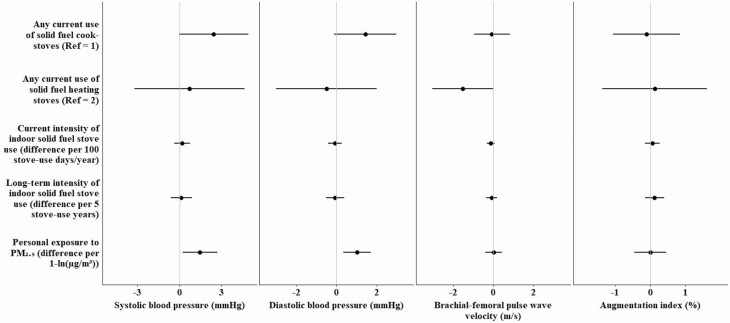
Associations of household fuel use and personal exposure to PM_2.5_ with blood pressure and arterial stiffness.Reference groups: 1 = current exclusive use of clean fuel cookstoves; 2 = current exclusive use of clean fuel heating stoves. Mixed effects regression models with participant- and village-specific random intercepts adjusting for age, sex, income, waist circumference, height (AIx, bfPWV), smoking status, secondhand smoke exposure, alcohol consumption, physical activity, 24-h urinary sodium, time of day of BP measurement (PM_2.5_ models only), and total/HDL cholesterol ratio were used. Results not shown for no heating stoves group (*n* = 19). Personal exposure to PM_2.5_ refers to the average season-specific exposures to PM_2.5_ over 2 consecutive days during each campaign. Point estimates are available on Supplementary Table S3 online.

**Figure 2.**
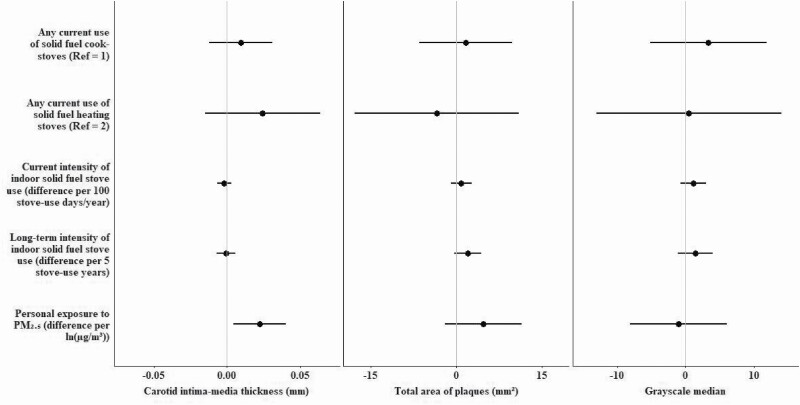
Associations of household fuel use and personal exposure to PM_2.5_ with atherosclerosis and plaques. Reference groups: 1 = current exclusive use of clean fuel cookstoves; 2 = current exclusive use of clean fuel heating stoves. Mixed effects regressions models with village-specific random intercepts adjusting for age, sex, yearly household income, waist circumference, alcohol consumption, smoking status, secondhand smoke exposure, physical activity, and total/HDL cholesterol ratio were used. Personal exposure to PM_2.5_ refers to estimated yearly exposure based on time-weighted averages of season-specific measurements of personal exposure to PM_2.5_ (7 nonheating and 5 heating months). Point estimates are available on Supplementary Table S3 online.

Current use of solid fuel cookstoves was associated with higher BP (systolic: 2.4 mm Hg, 95% CI: −0.4, 4.9; diastolic: 1.4 mm Hg, 95% CI: −0.1, 3.0) compared with exclusive clean fuel cooking (Figure 1), though both confidence intervals included zero. We did not find consistent associations between current use of solid fuel heaters and intensity of stove use with BP or between fuel use and arterial stiffness, except for an inverse association between current use of solid fuel heaters and lower bfPWV (−1.5 m/s, 95% CI: −3.0, 0.0). Current and long-term solid fuel use and greater intensity of use were all associated with greater CIMT and features of plaques, but the coefficients were small and confidence intervals included zero (Figure 2).

Fuel use-vascular associations were higher among older participants (Supplementary Figure S2 online), but overall age was not a strong modifier. We also observed associations between solid fuel cookstove use and higher BP among northern China participants compared with those in (southern) Guangxi (interaction *P* < 0.05) (Supplementary Figure S3 online). Associations between current solid fuel cooking and BP were higher in summer (systolic: 3.6 mm Hg, 95% CI: 0.9, 6.3; and, diastolic: 3.0 mm Hg, 95% CI: 1.2, 4.8) than winter (systolic: 0.0 mm Hg, 95% CI: −2.6, 2.7; diastolic: −0.2 mm Hg, 95% CI: −1.9, 1.5), while bfPWV was lower in the summer (−1.0 m/s, 95% CI: −2.1, 0.0; vs. winter: 0.4, 95% CI: −0.6. 1.4). Sex and use of antihypertensive medication did not modify the associations.

### Associations of personal exposure to PM_2.5_ with blood pressure and vascular function

Estimated yearly exposure to PM_2.5_ (per 1 − ln(µg/m^3^)) was associated with higher BP (systolic: 1.5 mm Hg, 95% CI: 0.2, 2.7; diastolic: 1.0, 95% CI: 0.4, 1.7) (Figure 1) and with greater CIMT (0.02 mm, 95% CI:0.00, 0.04), and total plaque area (4.7 mm^2^, 95% CI: −2.0, 11.5) in multivariable models (Figure 2). Exposure to PM_2.5_ was not associated with arterial stiffness (bfPWV, AIx) or GSM in the main analysis. Age did not strongly modify the PM_2.5_–vascular relationships (Supplementary Figure S2 online), except for GSM which was inversely associated with PM_2.5_ only in older participants (interaction *P* < 0.01).

### Sensitivity analyses

Limiting our analysis of current stove use to only indoor stoves attenuated the associations between stove use and BP, likely due to greater exposure misclassification (Supplementary Table S3 online). Additional sensitivity analyses did not change our overall findings, including adjustment for hypertension status or excluding those with diabetes, both which had little effect on our point estimates. Additional adjusting for heating fuel in models with cooking fuel as the exposure and vice versa also did not change our overall findings (Supplementary Table S4 online).

## DISCUSSION

In this multi-provincial study of Chinese adults, exposure to household air pollution was associated with higher BP and with greater CIMT and total area of carotid plaques. The associations between household air pollution and arterial stiffness were mostly null, with the exception of lower bfPWV in users of solid fuel heaters.

Our study has a number of important strengths. Our relatively large sample included men, who comprise 35% of household air pollution-attributable cardiovascular disease-related burden^20^ but are understudied.^21^ We controlled for a comprehensive set of confounders including socioeconomic status and dietary indicators that were not considered in previous studies.^11,12,14^ We measured up to 96 hours of exposure to PM_2.5_ across seasons, allowing us to estimate yearly averages, which are relevant for long-term outcomes like CIMT and plaques. Finally, our outcomes reflect different domains of functional and structural vascular damage, and are recognized risk factors for cardiovascular and renal disease.

The magnitude of associations between household air pollution and BP in our study (1–3 mm Hg) overlap with global studies of primary solid fuel users (0.6–4 mm Hg)^22–24^ and exposure–response studies in China, India, and Honduras (1–4 mm Hg per 1 ln(µg/m^3^) increase in PM_2.5_).^25–28^ Household air pollution was not associated with arterial stiffness in our study, with the exception of an unexpected finding of lower bfPWV among users of solid fuel heaters. Previously, our group found a null association between household air pollution and arterial stiffness, but outdoor air pollution has been linked with vascular damage through ventricular repolarization-induced myocardial vulnerability and arrhythmias.^25,29^ A possible explanation is residual confounding by home (indoor) temperature, which is associated with BP and arterial stiffness.^30^

The PM_2.5_-CIMT coefficient [0.02 mm per 1 ln(µg/m^3^) increase in PM_2.5_] in our study is similar to CIMT differences between biomass and clean fuel users in Peru (0.03 mm)^31^ and Nigeria (0.04 mm).^6^ Our CIMT results are supported by our finding that PM_2.5_ and solid fuel stove use were associated with a greater total plaque area, which are consistent with a study of biomass use and prevalence of atherosclerotic plaque in Peru.^31^ Outdoor PM_2.5_ has also been linked with greater carotid plaque (HR: 1.78, 95% CI: 1.55, 2.03) in a cohort of previously carotid plaque-free individuals living in Shijiazhuang, China.^32^ Our study provides a new dimension to the existing literature on air pollution and atherosclerosis, which is mostly comprised studies in European and North American populations exposed to much lower air pollution (e.g., 0.02 mm per 10 µg/m^3^, with PM_2.5_ ranging from 13 to 23 µg/m^3^).^33^

Our finding linking lower GSM, an indicator of plaque instability and rupture risk,^17^ with higher PM_2.5_ in older participants is novel but should be interpreted with caution. Greater than 90% of the women in our study sample were postmenopausal and are at greater risk for vascular remodeling.^34^ As such, older participants (≥63 years) were more likely to have plaques than younger participants (80% vs. 69%), though no significant interactions between longer exposures and age were observed. Plaques can become more vulnerable to rupture after exposure to PM_2.5_ via blood thrombogenicity and the promotion of foam cell formation.^35^ However, this finding was not supported by our fuel use analysis, and our ultrasound images were not designed for advanced plaque tissue characterization.

Differences in underlying health and cardiovascular risk profiles of participants from northern vs. southern China could explain the larger associations in our northern participants. Northern China has higher overall cardiovascular risk including stroke.^36^ Differences in fuel types and PM_2.5_ composition may also differentially affect hypertension and vascular function of participants in northern vs. southern China.^37^ Biomass stoves were used in all sites, whereas coal was more common in the north. However, we could not differentiate between fuel types in our analysis as most northern households using biomass also used coal. Notably, restricting our fuel use exposure measure to only indoor stoves attenuated the associations between stove use and BP. Air exchanges between the outdoor and indoor environments are high in rural China^38^ such that pollution from outdoor stoves could still impact personal exposures. It is possible that excluding outdoor stoves introduced exposure misclassification which led to bias toward the null.

Based on our multivariable models, the difference in BP and vascular outcomes for participants in the 90th vs. 10th percentile of PM_2.5_ exposure are 2.5 mm Hg systolic BP, 1.8 mm Hg diastolic BP, 0.03 mm CIMT, and 6.6 mm^2^ total area of plaques. Although associations of this magnitude are relatively small, they translate into substantial clinically meaningful reduction in BP at the population level. For example, 10 mm Hg higher systolic BP is associated with a 21% increase in cardiovascular mortality in Chinese adults.^39^ Even a 2 mm Hg reduction in systolic BP can reduce coronary heart disease by 4%, and stroke mortality by 6%,^40^ and a 2 mm Hg reduction in diastolic BP is associated with a 14% reduction in stroke risk, and 6% reduction in coronary heart disease in 35–64-year US men.^41^ Our study did not capture the change in various indicators of vascular damage due to household air pollution given the relatively short follow-up (<9 months) between each campaign, but a 0.1 mm decrease in CIMT translates to a 15% decrease in risk myocardial infraction and a 17% decrease in stroke.^42^

Our study has several limitations to consider for future studies. Stove use was self-reported and retrospectively assessed, and thus subject to recall bias. The mostly null findings for long-term solid fuel use, in contrast to more consistent associations with current practices, may be due to exposure misclassification. Measurement error in personal exposure to PM_2.5_ is also likely given the large within-individual variability in exposure among our participants (σ ^2^ = 0.80),^14^ though this error most likely nondifferential and would bias our results toward the null. Residual confounding is possible in this observational study. Our repeated-measures design accounted for nonvarying factors, and we measured most known BP and vascular risk factors, including behavioral and socioeconomic factors not previously measured. Though we were unable to measure home temperature due to study logistics, which may have confounded some wintertime associations. Further, the findings from our repeated measures analysis should be interpreted with caution as we obtained only one measurement of some time-varying covariates like lipids and did not collect information on indoor temperature or BP medication compliance. Finally, given that our study is an observational and cross-sectional study, it is limited in its ability to identify causal effects. Future studies in this cohort can assess if household air pollution affects the development of hypertension or the progression of atherosclerosis and plaque instability.

These findings add to a small but rapidly growing body of evidence that household air pollution is associated with subclinical cardiovascular outcomes including higher blood pressure, and our study contributes to the relatively limited exposure–response data on personal exposure to household air pollution and the severity of atherosclerosis and vascular disease, particularly for men. China’s cardiovascular burden exceeds the global average, and is expected to increase by 3.4 million excess deaths by 2030 as its population ages.^43^ Reduction of elevated BP can effectively slow the progression of end-stage renal and cardiovascular disease.^44^ Population interventions that reduce BP and the prevalence of hypertension are urgently needed, and our study contributes to evidence that clean household energy could be part of a comprehensive hypertension prevention strategy.

## Supplementary Material

hpab141_suppl_Supplementary_Material

## Data Availability

Corresponding authors may be contacted for study data.
