# Supplementary material for: Household Air Pollution and Blood Pressure, Vascular Damage, and Subclinical Indicators of Cardiovascular Disease in Older Chinese Adults
Source: Am J Hypertens. 2021 Sep 10;35(2):121–31. doi: 10.1093/ajh/hpab141 (PMC8807175; doi:10.1093/ajh/hpab141)
Supplement: hpab141_suppl_Supplementary_Material [file hpab141_suppl_supplementary_material.docx]

Table S1. Variables with missing data (n (%) or mean (SD)).

|  | **Men** | | | **Women** | | |
| --- | --- | --- | --- | --- | --- | --- |
|  | **Shanxi** | **Beijing** | **Guangxi** | **Shanxi** | **Beijing** | **Guangxi** |
|  | **(n=130)** | **(n=105)** | **(n=102)** | **(n=154)** | **(n=141)** | **(n=121)** |
| Yearly household income (CYN) |  |  |  |  |  |  |
| <2,500 | 34 (26.2%) | 3 (2.9%) | 1 (1.0%) | 32 (20.8%) | 2 (1.4%) | 6 (5.0%) |
| 2,500 to 4,999 | 9 (6.9%) | 8 (7.6%) | 1 (1.0%) | 9 (5.8%) | 13 (9.2%) | 1 (0.8%) |
| 5,000 to 9,999 | 11 (8.5%) | 21 (20.0%) | 1 (1.0%) | 15 (9.7%) | 20 (14.2%) | 1 (0.8%) |
| 10,000 to 19,999 | 20 (15.4%) | 31 (29.5%) | 8 (7.8%) | 20 (13.0%) | 21 (14.9%) | 5 (4.1%) |
| 20,000 to 34,999 | 18 (13.8%) | 17 (16.2%) | 11 (10.8%) | 34 (22.1%) | 32 (22.7%) | 25 (20.7%) |
| ≥35,000 | 14 (10.8%) | 21 (20.0%) | 73 (71.6%) | 11 (7.1%) | 42 (29.8%) | 75 (62.0%) |
| Missing | 24 (18.5%) | 4 (3.8%) | 7 (6.9%) | 33 (21.4%) | 11 (7.8%) | 8 (6.6%) |
| Yearly household income (CYN) |  |  |  |  |  |  |
| <20,000 | 74 (56.9%) | 63 (60.0%) | 11 (10.8%) | 76 (49.4%) | 56 (39.7%) | 13 (10.7%) |
| ≥20,000 | 32 (24.6%) | 38 (36.2%) | 84 (82.4%) | 45 (29.2%) | 74 (52.5%) | 100 (82.6%) |
| Missing | 24 (18.5%) | 4 (3.8%) | 7 (6.9%) | 33 (21.4%) | 11 (7.8%) | 8 (6.6%) |
| Waist circumference (cm) |  |  |  |  |  |  |
| Mean (SD) | 89.5 (11.7) | 90.5 (7.9) | 83.3 (9.5) | 88.2 (8.9) | 89.3 (9.4) | 82.3 (8.9) |
| Missing | 5 (3.8%) | 2 (1.9%) | 0 (0%) | 2 (1.3%) | 7 (5.0%) | 0 (0%) |
| Body mass index (kg/m^2^) |  |  |  |  |  |  |
| Mean (SD) | 25.2 (3.9) | 25.6 (3.0) | 23.3 (3.6) | 26.4 (3.4) | 26.9 (3.8) | 23.5 (3.5) |
| Missing | 5 (3.8%) | 2 (1.9%) | 0 (0%) | 2 (1.3%) | 7 (5.0%) | 1 (0.8%) |
| Mean 24h urinary sodium excretion (mmol/day) |  |  |  |  |  |  |
| Mean (SD) | 221.5 (83.7) | 204.9 (89.4) | 126.0 (56.1) | 189.9 (65.4) | 175.0 (71.1) | 101.3 (45.6) |
| Missing | 6 (4.6%) | 4 (3.8%) | 0 (0%) | 4 (2.6%) | 11 (7.8%) | 1 (0.8%) |
| Total/HDL cholesterol ratio |  |  |  |  |  |  |
| Mean (SD) | 3.9 (1.0) | 3.9 (1.2) | 4.5 (5.7) | 3.9 (0.8) | 4.0 (1.0) | 3.7 (0.9) |
| Missing | 32 (24.6%) | 18 (17.1%) | 3 (2.9%) | 36 (23.4%) | 10 (7.1%) | 8 (6.6%) |
| CYN = Chinese Yuan, HDL = high-density lipoproteins, SD = standard deviation. Missing variables were imputed with the MICE package that uses the Markov Chain Monte Carlo (MCMC) method to assess the correlation structure of the data and imputes missing values for each incomplete variable 5 times by regression of incomplete variables on the other variables iteratively. We included the following other variables, age, sex, physical activity, alcohol consumption, smoking status, secondhand smoke exposure, occupation, education, and body mass index to generate values for the missing variables, income, waist circumference, urinary sodium, and blood lipids. | | | | | | |

Table S2. Characteristics of study participants by province and sex (n (%) or mean (SD)).

|  | **Men** | | | **Women** | | |
| --- | --- | --- | --- | --- | --- | --- |
|  | **Shanxi** | **Beijing** | **Guangxi** | **Shanxi** | **Beijing** | **Guangxi** |
|  | **(n=130)** | **(n=105)** | **(n=102)** | **(n=154)** | **(n=141)** | **(n=121)** |
| Age (years) | 62.3 (9.2) | 64.7 (7.3) | 62.6 (9.7) | 61.7 (8.3) | 62.4 (7.6) | 63.7 (9.4) |
| Highest educational attainment |  |  |  |  |  |  |
| No formal education | 6 (4.6%) | 10 (9.5%) | 4 (3.9%) | 23 (14.9%) | 49 (34.8%) | 22 (18.2%) |
| Primary school | 54 (41.5%) | 45 (42.9%) | 42 (41.2%) | 82 (53.2%) | 34 (24.1%) | 55 (45.5%) |
| Early high school, college, or above | 70 (53.8%) | 50 (47.6%) | 56 (54.9%) | 49 (31.8%) | 58 (41.1%) | 44 (36.4%) |
| Yearly household income (CYN) |  |  |  |  |  |  |
| <20,000 | 93 (71.5%) | 66 (62.9%) | 12 (11.8%) | 103 (66.9%) | 63 (44.7%) | 13 (10.7%) |
| ≥20,000 | 37 (28.5%) | 39 (37.1%) | 90 (88.2%) | 51 (33.1%) | 78 (55.3%) | 108 (89.3%) |
| Occupation |  |  |  |  |  |  |
| Agricultural | 91 (70.0%) | 91 (86.7%) | 14 (13.7%) | 118 (76.6%) | 106 (75.2%) | 18 (14.9%) |
| Retired or not currently employed | 20 (15.4%) | 5 (4.8%) | 70 (68.6%) | 35 (22.7%) | 31 (22.0%) | 92 (76.0%) |
| Non-agricultural | 19 (14.6%) | 9 (8.6%) | 18 (17.6%) | 1 (0.6%) | 4 (2.8%) | 11 (9.1%) |
| Tobacco smoking |  |  |  |  |  |  |
| Never | 9 (6.9%) | 16 (15.2%) | 24 (23.5%) | 141 (91.6%) | 141 (100%) | 121 (100%) |
| Past | 48 (36.9%) | 305 (33.3%) | 38 (37.3%) | 5 (3.2%) | 0 (0%) | 0 (0%) |
| Current | 73 (56.2%) | 54 (51.4%) | 40 (39.2%) | 8 (5.2%) | 0 (0%) | 0 (0%) |
| Secondhand smoke exposure |  |  |  |  |  |  |
| Never | 128 (98.5%) | 102 (97.1%) | 92 (90.2%) | 45 (29.2%) | 29 (20.6%) | 33 (27.3%) |
| Past | 0 (0%) | 0 (0%) | 2 (2.0%) | 39 (25.3%) | 42 (29.8%) | 40 (33.1%) |
| Current | 2 (1.5%) | 3 (2.9%) | 8 (7.8%) | 70 (45.5%) | 70 (49.6%) | 48 (39.7%) |
| Alcohol consumption (past year) |  |  |  |  |  |  |
| Never | 47 (36.2%) | 26 (24.8%) | 31 (30.4%) | 138 (89.6%) | 124 (87.9%) | 94 (77.7%) |
| Occasional (<1 drink per week) | 46 (35.4%) | 26 (24.8%) | 30 (29.4%) | 14 (9.1%) | 14 (9.9%) | 24 (19.8%) |
| Regular (≥1 drink per week) | 37 (28.5%) | 53 (50.5%) | 41 (40.2%) | 2 (1.3%) | 3 (2.1%) | 3 (2.5%) |
| Physical activity (frequency in past 3 months) |  |  |  |  |  |  |
| None | 59 (45.4%) | 78 (74.3%) | 60 (58.8%) | 57 (37.0%) | 91 (64.5%) | 91 (75.2%) |
| ≤2 times per week | 25 (19.2%) | 17 (16.2%) | 21 (20.6%) | 45 (29.2%) | 26 (18.4%) | 14 (11.6%) |
| ≥3 times per week | 46 (35.4%) | 10 (9.5%) | 21 (20.6%) | 52 (33.8%) | 24 (17.0%) | 16 (13.2%) |
| Hypertension (% yes) | 57 (43.8%) | 66 (62.9%) | 42 (41.2%) | 91 (59.1%) | 94 (66.7%) | 43 (35.5%) |
| Current use of anti-hypertensive medication (% yes) | 53 (40.8%) | 42 (40.0%) | 9 (8.8%) | 84 (54.5%) | 74 (52.5%) | 19 (15.7%) |
| Clinician-diagnosed diabetes (% yes) | 17 (13.1%) | 12 (11.4%) | 6 (5.9%) | 34 (22.1%) | 17 (12.1%) | 5 (4.1%) |
| Waist circumference (cm) | 89.4 (11.5) | 90.3 (8.14) | 83.3 (9.5) | 88.1 (8.9) | 89.1 (9.5) | 82.3 (8.9) |
| Body mass index (kg/m^2^) | 25.2 (3.9) | 25.5 (3.0) | 23.3 (3.6) | 26.5 (3.4) | 26.8 (3.8) | 23.5 (3.5) |
| Height (cm) | 167.1 (6.4) | 166.2 (6.1) | 162.2 (6.8) | 153.8 (6.4) | 154.3 (5.7) | 150.7 (5.9) |
| Mean 24h urinary sodium excretion (mmol/day) | 220.1 (83.8) | 205.7 (91.6) | 126.0 (56.1) | 189.9 (65.0) | 175.4 (71.2) | 101.3 (45.4) |
| Total cholesterol (mmol/L) | 4.4 (0.9) | 4. 8 (1.1) | 5.5 (1.0) | 4.7 (0.9) | 5.1 (1.1) | 5. 8 (1.1) |
| LDL (mmol/L) | 2.5 (0.7) | 2.8 (0.9) | 3.4 (0.8) | 2.7 (0.8) | 3.0 (1.0) | 3.7 (1.0) |
| HDL (mmol/L) | 1.1 (0.3) | 1.3 (0.3) | 1.4 (0.4) | 1.2 (0.3) | 1.3 (0.3) | 1.6 (0.3) |
| Total/HDL cholesterol ratio | 4.0 (1.0) | 3.8 (1.1) | 4.5 (5.6) | 3.9 (0.9) | 4.0 (1.0) | 3.7 (0.9) |
| CYN = Chinese Yuan, Combined physical activity = exercise and farm-based physical activity domains, Hypertension diagnosis = current use of anti-hypertensive medication, systolic (≥140 mmHg) or diastolic (≥90 mmHg) blood pressure, HDL = high-density lipoproteins, SD = standard deviation. | | | | | | |

Table S3. Results from sensitivity analyses for multivariable models with exposures to (a) any use of solid fuel cookstoves, (b) any use of solid fuel heating stoves (northern China only), and (c) estimated yearly average exposure to PM_2.5_.

|  | **(a) exposure - any use of solid fuel cookstoves^1^** | | | | | | |
| --- | --- | --- | --- | --- | --- | --- | --- |
| **Blood pressure and vascular outcomes** | | **Results from main analysis**  **(n=753)** | | **Indoor solid fuel stove use only**  **(n=753)** | **Excluding participants with diabetes**  **(n=662)** | | **Additional adjustment for hypertension**  **(n=726)** |
| Systolic blood pressure (mmHg) | | 2.4 (-0.4, 4.9) | | 1.1 (-1.4, 3.5) | 1.9 (-0.7, 4.5) | | - |
| Diastolic blood pressure (mmHg) | | 1.4 (-0.1, 3.0) | | 0.1 (-1.4, 1.6) | 1.4 (-0.3, 3.1) | | - |
| Brachial-femoral pulse wave velocity (m/s) | | -0.1 (-1.0, 0.8) | | -0.2 (-1.0, 0.7) | -0.1 (-1.1, 0.8) | | 0.0 (-0.9. 0.9) |
| Augmentation index (%) | | -0.1 (-1.0, 0.9) | | -0.3 (-1.3, 0.6) | -0.2 (-1.2, 0.9) | | -0.1 (-1.1, 0.8) |
| Carotid intima-media thickness (mm) | | 0.01 (-0.01, 0.03) | | -0.01 (-0.03, 0.01) | 0.01 (-0.01, 0.03) | | 0.01 (-0.01, 0.03) |
| Total area of plaques (mm^2^) | | 1.7 (-6.5, 9.8) | | 3.3 (-4.8, 11.1) | 1.7 (-7.2, 10.7) | | 1.7 (-6.3, 9.7) |
| Grayscale median | | 3.4 (-5.1, 11.9) | | 5.1 (-3.1, 13.3) | 4.1 (-4.9, 13.2) | | 3.7 (-4.8, 12.2) |
|  | **(b) exposure - any use of solid fuel heating stoves^2^** | | | | | | |
| **Blood pressure and vascular outcomes** | | **Results from main analysis**  **(n=530)** | **Indoor solid fuel stove use only**  **(n=530)** | | | **Excluding participants with diabetes**  **(n=450)** | **Additional adjustment for hypertension**  **(n=505)** |
| Systolic blood pressure (mmHg) | | 0.7 (-3.2, 4.6) | 0.5 (-2.5, 3.5) | | | 0.5 (-3.6, 4.6) | - |
| Diastolic blood pressure (mmHg) | | -0.5 (-3.0, 2.0) | -0.3 (-2.1, 1.6) | | | -0.3 (-3.1, 2.5) | - |
| Brachial-femoral pulse wave velocity (m/s) | | -1.5 (-3.0, -0.0) | -0.8 (-1.9, 0.3) | | | -0.9 (-2.4, 0.6) | -1.3 (-2.8, 0.2) |
| Augmentation index (%) | | 0.1 (-1.4, 1.6) | -0.1 (-1.3, 1.0) | | | 0.6 (-1.1, 2.2) | 0.2 (-1.4, 1.7) |
| Carotid intima-media thickness (mm) | | 0.02 (-0.02, 0.0.6) | 0.00 (-0.03, 0.03) | | | 0.03 (-0.02, 0.0.7) | 0.03 (-0.01, 0.07) |
| Total area of plaques (mm^2^) | | -3.4 (-17.8, 11.0) | 2.3 (-8.1, 12.8) | | | 0.4 (-15.9, 16.7) | -2.5 (-16.8, 11.9) |
| Grayscale median | | 0.5 (-13.0, 14.1) | 2.3 (-8.6, 13.1) | | | 1.9 (-12.5, 16.3) | 0.5 (-13.2, 14.2) |
|  | **(c) exposure - personal exposure to PM_2.5_ (per 1-ln(μg/m^3^))** | | | | | | |
| **Blood pressure and vascular outcomes** | | **Results from main analysis**  **(n=747)** | |  | **Excluding participants with diabetes**  **(n=657)** | | **Additional adjustment for hypertension**  **(n=721)** |
| Systolic blood pressure (mmHg) | | 1.5 (0.2, 2.7) | |  | 1.5 (0.2, 2.8) | | - |
| Diastolic blood pressure (mmHg) | | 1.0 (0.4, 1.7) | |  | 1.2 (0.5, 1.9) | | - |
| Brachial-femoral pulse wave velocity (m/s) | | 0.0 (-0.4, 0.4) | |  | 0.1 (-0.3, 0.5) | | -0.0 (-0.4, 0.4) |
| Augmentation index (%) | | 0.0 (-0.5, 0.5) | |  | -0.0 (-0.5, 0.5) | | 0.0 (-0.5, 0.5) |
| Carotid intima-media thickness (mm) | | 0.02 (0.00, 0.04) | |  | 0.02 (0.00, 0.04) | | 0.02 (0.00, 0.04) |
| Total area of plaques (mm^2^) | | 4.7 (-2.0, 11.5) | |  | 5.1 (-2.1, 12.4) | | 4.6 (-2.1, 11.3) |
| Grayscale median | | -1.0 (-8.1, 6.1) | |  | -2.8 (-10.3, 4.7) | | -0.8 (-7.9, 6.3) |
| CCA = common carotid artery; Bulb = bifurcation; ICA = internal carotid artery; and, ECA = external carotid artery. ^1^Reference = exclusive use of clean fuel cookstoves, and ^2^Reference = exclusive use of clean fuel heating stoves. | | | | | | | |

Table S4. Associations of household fuel use and blood pressure with additional adjustments

|  | **Models** | **Systolic blood pressure (mmHg)** | **Diastolic blood pressure (mmHg)** |
| --- | --- | --- | --- |
|  |  | **Difference (95% CI)** | **Difference (95% CI)** |
| **Any use of solid fuel cookstoves^1^** | Model 1 | 3.7 (0.9, 6.6) | 2.9 (1.1, 4.7) |
|  | Model 1 + Heating stoves | 3.7 (0.8, 6.6) | 3.0 (1.1, 4.8) |
| **Any use of solid fuel heating stoves^2^** | Model 1 | 0.7 (-3.2, 4.6) | -0.5 (-3.0, 2.0) |
|  | Model 1 + Cookstoves | 0.7 (-4.7, 6.0) | -2.2, (-5.8, 1.3) |
| Reference groups: ^1^exclusive use of clean fuel cookstoves; ^2^exclusive use of clean fuel heating stoves. Model 1 = adjusted for age, sex, yearly household income, waist circumference, alcohol consumption, smoking status, secondhand smoke exposure, alcohol consumption, physical activity, and total/HDL cholesterol ratio. | | | |

Table S5. Effect modification by median age for associations of household fuel use and personal exposure to PM_2.5_ with blood pressure and vascular outcomes.

| **Household air pollution exposures** | **Median age** | **Systolic blood pressure (mmHg)** | **Diastolic blood pressure (mmHg)** | **Brachial-femoral pulse wave velocity (m/s)** | **Augmentation index (%)** | **Carotid intima-media thickness (mm)** | **Total area of plaques (mm^2^)** | **Grayscale median** |
| --- | --- | --- | --- | --- | --- | --- | --- | --- |
| **Any current use of solid fuel cookstoves^1^** |  |  |  |  |  |  |  |  |
|  | <63y | 0.5 (-2.9, 3.9) | 0.6 (-1.5, 2.7) | 0.0 (-1.2, 1.2) | -0.5 (-1.8, 0.8) | 0.00 (-0.03, 0.03) | -2.7 (-13.7, 8.4) | 7.5 (-4.5, 19.5) |
|  | ≥63y | 4.3 (0.8, 7.7) | 2.3 (0.2, 4.4) | -0.2 (-1.4, 1.1) | 0.3 (-1.0, 1.6) | 0.02 (-0.01, 0.05) | 6.0 (-5.0, 17.1) | -0.1 (-11.3, 11.0) |
| **Any current use of current solid fuel heating stoves^2^** |  |  |  |  |  |  |  |  |
|  | <63y | -0.2 (-5.6, 5.3) | -1.1 (-4.6, 2.3) | -1.0 (-3.1, 1.1) | -0.6 (-2.7, 1.5) | 0.03 (-0.02, 0.09) | -8.9 (-28.2, 10.4) | 1.0 (-17.0, 18.9) |
|  | ≥63y | 2.0 (-3.4, 7.4) | 0.2 (-3.2, 3.5) | -1.9 (-4.0, 0.1) | 0.7 (-1.4, 2.8) | 0.02 (-0.04, 0.07) | 3.0 (-16.3, 22.2) | -0.0 (-19.0, 19.0) |
| **Current intensity of indoor solid fuel use (per 100 stove-use days/year)** |  |  |  |  |  |  |  |  |
|  | <63y | -0.0 (-0.8, 0.8) | -0.1 (-0.6, 0.4) | -0.0 (-0.3, 0.3) | 0.1 (-0.2, 0.4) | -0.00 (-0.01, 0.00) | -1.2 (-3.8, 1.5) | 2.1 (-0.7, 5.0) |
|  | ≥63y | 0.4 (-0.4, 1.1) | -0.1 (-0.5, 0.4) | -0.2 (-0.5, 0.1) | 0.0 (-0.3, 0.3) | -0.00 (-0.01, 0.01) | 2.4 (0.1, 4.8) | 0.6 (-1.8, 2 |
| **Long-term intensity of indoor solid fuel use (per 5 stove-use year)** |  |  |  |  |  |  |  |  |
|  | <63y | -0.1 (-1.2, 0.9) | -0.2 (-0.9, 0.4) | -0.0 (-0.4, 0.3) | 0.2 (-0.2, 0.6) | -0.00 (-0.01, 0.01) | 0.3 (-3.1, 3.7) | 2.5 (-1.2, 6.2) |
|  | ≥63y | 0.4 (-0.6, 1.4) | 0.1 (-0.6, 0.7) | -0.1 (-0.5, 0.3) | 0.1 (-0.3, 0.5) | 0.00 (-0.01, 0.01) | 3.6 (0.4, 6.8) | 0.6 (-2.8, 4.0) |
| **Personal exposure to PM_2.5_ (per 1-ln(μg/m^3^))** |  |  |  |  |  |  |  |  |
|  | <63y | 1.8 (0.1, 3.5) | 1.4 (0.4, 2.3) | 0.0 (-0.6, 0.6) | -0.1 (-0.7, 0.5) | 0.02 (-0.00, 0.05) | 5.7 (-3.9, 15.2) | 10.4 (-0.3, 21.0) |
|  | ≥63y | 1.2 (-0.5, 3.0) | 0.7 (-0.2, 1.7) | 0.0 (-0.6, 0.6) | 0.1 (-0.6, 0.7) | 0.02 (-0.01, 0.04) | 4.1 (-4.8, 13.0) | -8.5 (-17.3, 0.3) |
| ^1^Reference = current exclusive use of clean fuel cookstoves, and ^2^Reference = current exclusive use of clean fuel heating stoves. | | | | | | | | |

Table S6. Effect modification by geographic region for associations of household fuel use and personal exposure to PM_2.5_ with blood pressure and vascular outcomes.

| **Household air pollution exposures** | **Geographic region** | **Systolic blood pressure (mmHg)** | **Diastolic blood pressure (mmHg)** | **Brachial-femoral pulse wave velocity (m/s)** | **Augmentation index (%)** | **Carotid intima-media thickness (mm)** | **Total area of plaques (mm^2^)** | **Grayscale median** |
| --- | --- | --- | --- | --- | --- | --- | --- | --- |
| **Any current use of solid fuel cookstoves^1^** |  |  |  |  |  |  |  |  |
|  | North | 3.7 (0.9, 6.6) | 2.9 (1.1, 4.7) | -0.2 (-1.3, 0.9) | 0.2 (-1.0, 1.3) | 0.01 (-0.02, 0.03) | 0.8 (-9.3, 10.8) | 0.8 (-9.6, 11.1) |
|  | South | -2.0 (-6.9, 2.9) | -2.3 (-5.3, 0.6) | 0.2 (-1.5, 1.8) | -0.7 (-2.4, 1.1) | 0.01 (-0.02, 0.05) | 3.8 (-10.0, 17.5) | 9.0 (-5.6, 23.5) |
| **Any current use of current solid fuel heating stoves^2^** |  |  |  |  |  |  |  |  |
|  | North | 0.7 (-3.2, 4.6) | -0.5 (-3.0, 2.0) | -1.5 (-3.0, -0.0) | 0.1 (-1.4, 1.6) | 0.02 (-0.02, 0.06) | -3.4 (-17.8, 11.0) | 0.5 (-13.1, 14.1) |
|  | South | - | - | - | - | - | - | - |
| **Current intensity of indoor solid fuel use (per 100 stove-use days/year)** |  |  |  |  |  |  |  |  |
|  | North | 0.5 (-0.2, 1.1) | 0.1 (-0.3, 0.5) | -0.2 (-0.4, 0.1) | 0.1 (-0.1, 0.4) | -0.00 (-0.01, 0.00) | 0.9 (-1.4, 3.3) | 0.4 (-2.0, 2.8) |
|  | South | -0.6 (-1.6, 0.4) | -0.4 (-1.1, 0.2) | -0.0 (-0.4, 0.3) | -0.0( -0.4, 0.3) | -0.00 (-0.01, 0.01) | 0.9 (-2.1, 3.8) | 2.6 (-0.6, 5.7) |
| **Long-term intensity of indoor solid fuel use (per 5 stove-use year)** |  |  |  |  |  |  |  |  |
|  | North | 0.3 (-0.5, 1.2) | -0.1 (-0.6, 0.5) | -0.1 (-0.4, 0.2) | 0.2 (-0.1, 0.5) | -0.00 (-0.01, 0.01) | 2.4 (-0.5, 5.4) | 0.3 (-2.9, 3.4) |
|  | South | -0.2 (-1.6, 1.2) | -0.1 (-0.9, 0.7) | -0.0 (-0.5, 0.5) | -0.1 (-0.6, 0.4) | -0.00 (-0.01, 0.01) | 0.8 (-3.2, 4.8) | 3.4 (-0.9, 7.6) |
| **Personal exposure to PM_2.5_ (per 1-ln(μg/m^3^))** |  |  |  |  |  |  |  |  |
|  | North | 1.7 (0.4, 3.0) | 1.1 (0.4, 1.8) | 0.0 (-0.4, 0.5) | -0.2 (-0.6, 0.3) | 0.02 (-0.00, 0.04) | 2.2 (-5.9, 10.2) | -1.8 (-10.2, 6.6) |
|  | South | -0.5 (-4.8, 3.8) | 0.7 (-1.9, 3.3) | 0.2 (-1.2, 1.7) | 1.3 (-0.2, 2.9) | 0.03 (-0.00, 0.06) | 10.2 (-1.8, 22.2) | -0.5 (-13.9, 12.9) |
| ^1^Reference = current exclusive use of clean fuel cookstoves, and ^2^Reference = current exclusive use of clean fuel heating stoves. | | | | | | | | |


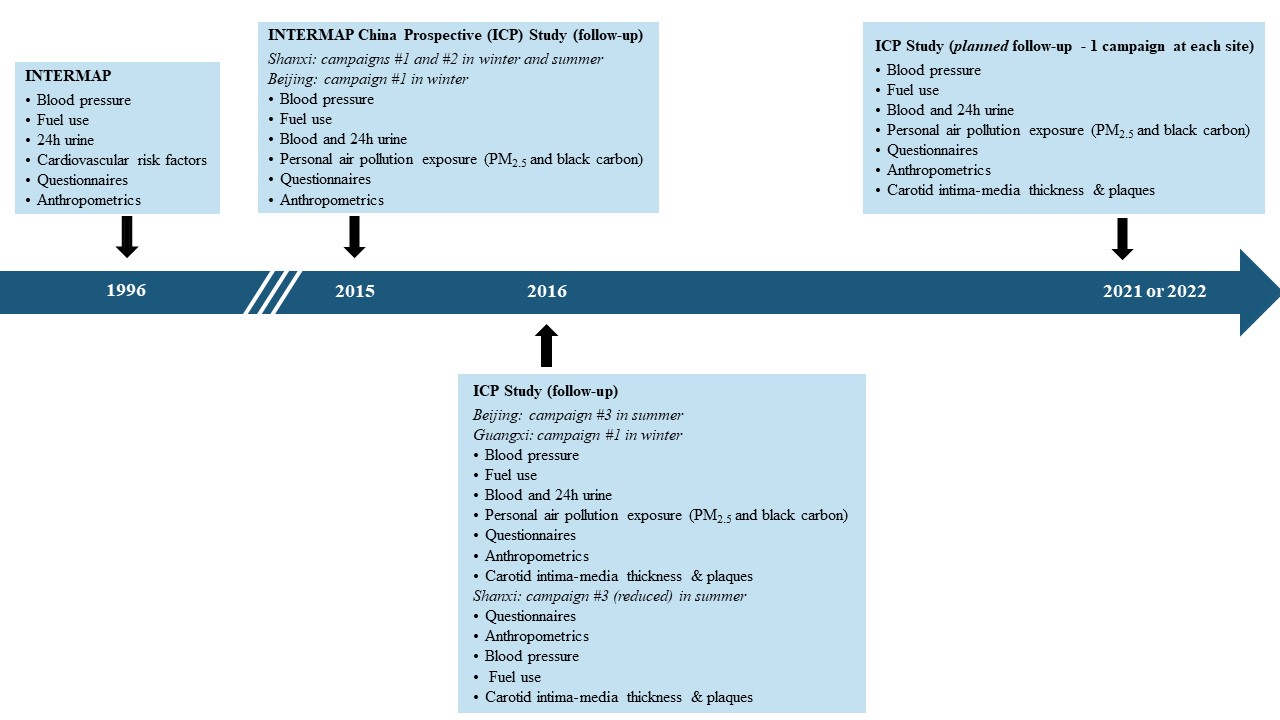


**Figure S1.** Timelines of the INTERMAP China Prospective Study.


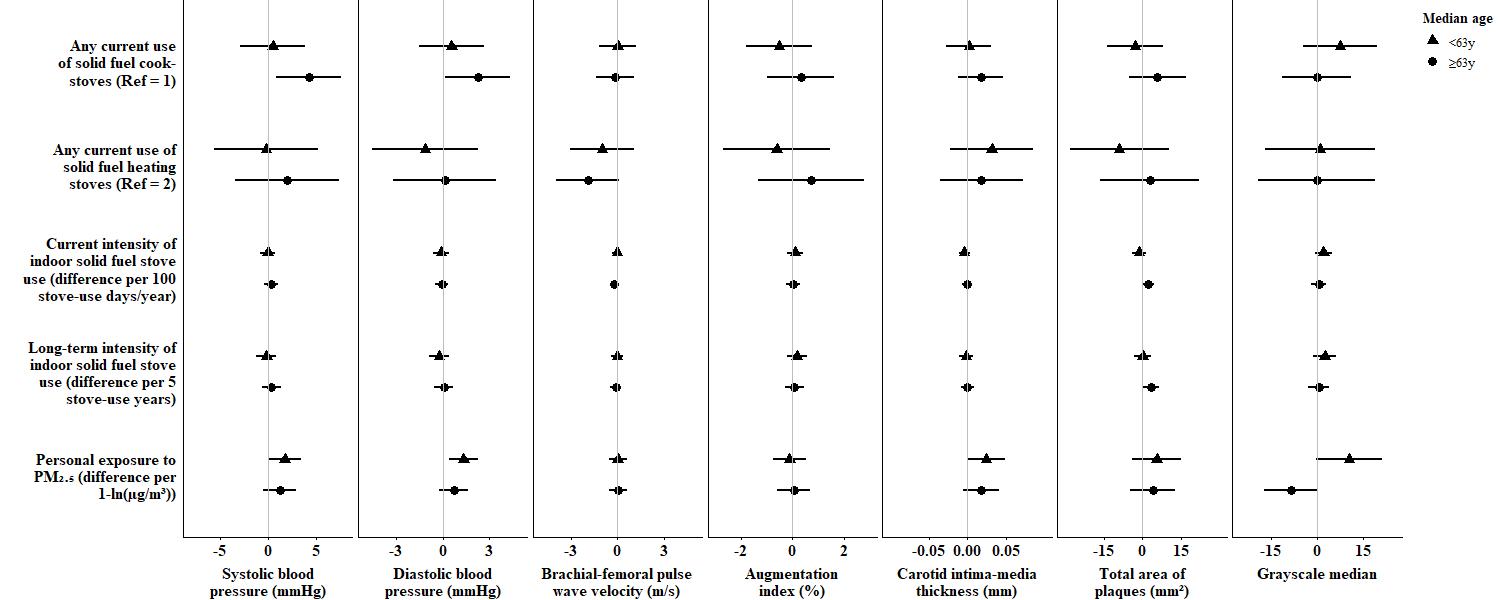


**Figure S2.** Associations of household fuel use and personal exposure to PM_2.5_ with blood pressure and vascular outcomes by median age.

Point estimates are available on Table S5.

**
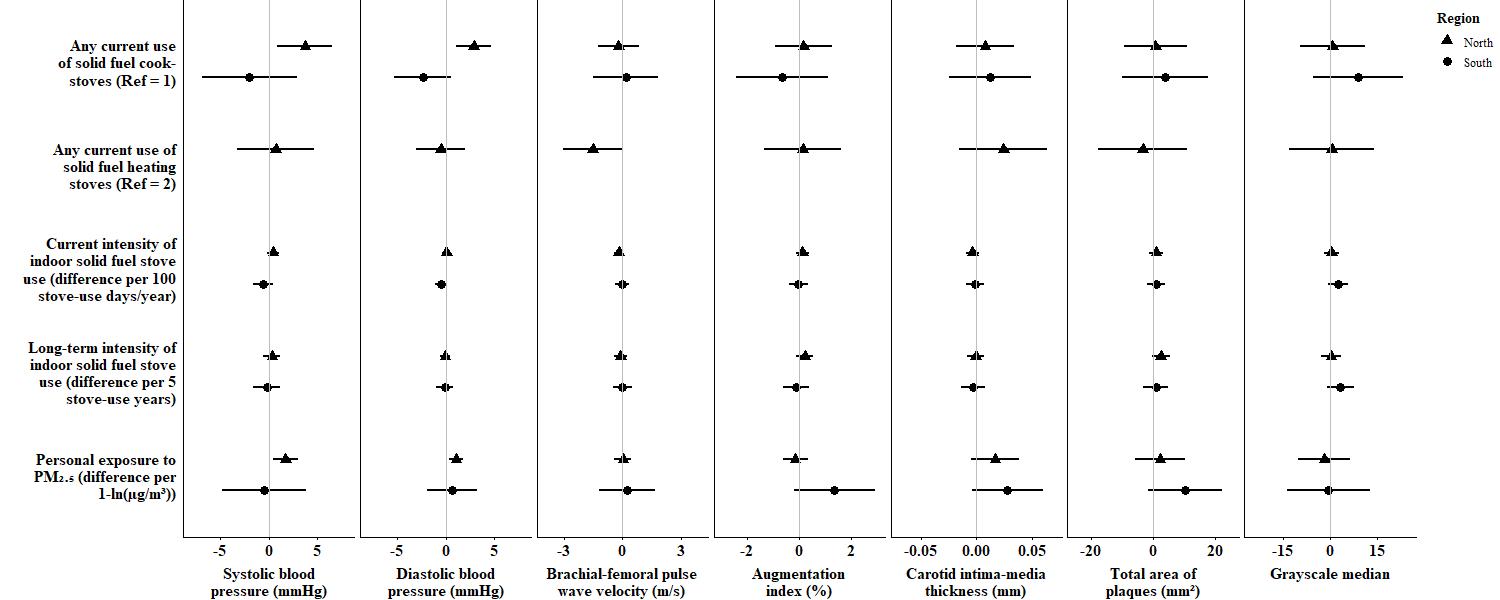
**

**Figure S3**. Associations of household fuel use and personal exposure to PM_2.5_ with blood pressure and vascular outcomes by geographic region.

North = Beijing and Shanxi; and South = Guangxi. Point estimates are available on Table S6.
